# MS-Net: a novel lightweight and precise model for plant disease identification

**DOI:** 10.3389/fpls.2023.1276728

**Published:** 2023-10-27

**Authors:** Siyu Quan, Jiajia Wang, Zhenhong Jia, Mengge Yang, Qiqi Xu

**Affiliations:** ^1^ School of Computer Science and Technology, Xinjiang University, Urumqi, China; ^2^ Xinjiang Uygur Autonomous Region Signal Detection and Processing Key Laboratory, Xinjiang University, Urumqi, China

**Keywords:** deep learning, plant disease recognition, convolutional neural network (CNN), transfer learning, lightweight networks

## Abstract

The rapid development of image processing technology and the improvement of computing power in recent years have made deep learning one of the main methods for plant disease identification. Currently, many neural network models have shown better performance in plant disease identification. Typically, the performance improvement of the model needs to be achieved by increasing the depth of the network. However, this also increases the computational complexity, memory requirements, and training time, which will be detrimental to the deployment of the model on mobile devices. To address this problem, a novel lightweight convolutional neural network has been proposed for plant disease detection. Skip connections are introduced into the conventional MobileNetV3 network to enrich the input features of the deep network, and the feature fusion weight parameters in the skip connections are optimized using an improved whale optimization algorithm to achieve higher classification accuracy. In addition, the bias loss substitutes the conventional cross-entropy loss to reduce the interference caused by redundant data during the learning process. The proposed model is pre-trained on the plant classification task dataset instead of using the classical ImageNet for pre-training, which further enhances the performance and robustness of the model. The constructed network achieved high performance with fewer parameters, reaching an accuracy of 99.8% on the PlantVillage dataset. Encouragingly, it also achieved a prediction accuracy of 97.8% on an apple leaf disease dataset with a complex outdoor background. The experimental results show that compared with existing advanced plant disease diagnosis models, the proposed model has fewer parameters, higher recognition accuracy, and lower complexity.

## 1 Introduction

As the population grows, the demand for food will increase dramatically, and it is particularly important to minimize food losses due to pests and diseases, which not only reduce food production but also affect biodiversity, food prices and human health, while trying to increase yields (Ristaino et al., 2021; Sileshi and Gebeyehu, 2021). Early prevention and control of plant diseases can recover some of the agricultural economic losses and improve the yield and quality of agricultural production and food safety (Savary et al., 2019; Gold, 2021). Thus, the diagnosis and control of crop diseases are crucial for food production. The traditional method of diagnosing plant pests and diseases is the visual observation by plant protection specialists or people with experience in planting. However, this approach relies heavily on experience and subjective cognition and is prone to bias, which can lead to misdiagnosis. (Bai et al., 2018; Barbedo, 2018). Moreover, in some underdeveloped or remote areas, there is often a shortage of experts. Therefore, one kind of portable, fast, and accurate plant disease automatic identification system is significant for the timely diagnosis of crop diseases.

Currently, the automatic diagnosis of plant diseases primarily relies on computer vision (CV) techniques. The predominant methods in this field can be categorized into two groups: machine learning-based approaches and deep learning-based approaches (Saeed et al., 2021; Uguz and Uysal, 2021). The widely used machine learning methods are the Bayesian model (BM), k-nearest neighbor (KNN), support vector machine (SVM), decision tree (DT), random forest tree (RF), etc. (Liakos et al., 2018; Chen et al., 2020). Within deep learning-based methods, many outstanding architectures such as ResNet, Inception, and DenseNet have achieved excellent results in image classification tasks. (Szegedy et al., 2015; He et al., 2016; Huang et al., 2017). Machine learning has made significant progress in the field of plant disease and pest recognition. However, it has a high degree of subjectivity, heavily relies on manual feature selection, is time-consuming, and has low efficiency (Li et al., 2021; Albattah et al., 2022b). In comparison, using deep learning methods is simpler and more efficient.

Recent studies demonstrated the effectiveness and feasibility of deep learning in plant disease classification tasks (Abbas et al., 2021; Deng et al., 2021; Elaraby et al., 2022). The end-to-end training approach avoids the drawbacks of manual feature extraction. Although there are many advanced deep CNN models for crop disease diagnosis, it is still difficult to promote this method on a large scale. The key reason is that complex models lead to high computational costs, making it difficult to deploy on simple mobile devices. In the agricultural field, using complex laboratory equipment with GPUs restricted the application and promotion of artificial intelligence, as growers cannot undertake the additional costs brought by complex equipment (Karthik et al., 2020; Chen et al., 2022a; Hassan and Maji, 2022). Therefore, lightweight models with fewer parameters, faster training speeds, and higher accuracy are a more promising research trend (Atila et al., 2021), which can further promote the popularization of automatic crop disease diagnosis methods. To address the aforementioned challenges, this paper proposes an improved MobileNetV3, which has low parameter count, high accuracy, and short training cycles. Specifically, we added two skip connections after the first bneck layer of the original feature extraction network. The low-level features extracted by the first bneck layer are used to compensate for the 7th and 11th bneck layers, thereby enriching the input features of the higher layers. Moreover, to achieve better results, different weights are assigned to the input feature maps of the skip connection parts, and the improved whale optimization algorithm is used to automatically adjust the weight parameters. Compared to manual hyperparameter tuning, the automatic optimization algorithm saves a significant amount of time and results in better model performance. The improved whale optimization algorithm enhances the search capability for global optimal parameters and convergence speed. Secondly, the Bias loss replaces the standard cross-entropy loss function. The Bias loss function can reduce the errors caused by redundant features during the model learning process. Another reason for the superior performance of the method proposed in this paper is the abandonment of the traditional ImageNet pre-training dataset. The constructed network is pre-trained on a large-scale plant classification task dataset. Transfer learning on similar objects further enhances the performance of the model. We refer to the re-formed lightweight network as MS-Net, which is mainly used for crop disease recognition. Experimental results demonstrate the effectiveness and feasibility of the proposed method. Compared to other SOTA models in the research field, MS-Net achieves the highest accuracy with lower parameter count, computational complexity, and memory size. The main contributions of this study are as follows.

1. We propose a new lightweight network, MS-Net. This network uses MobileNetV3 as the feature extraction network, embeds skip connections into the network, and adjusts the loss function, thereby improving the model’s accuracy and convergence speed.
2. The improved WOA (Whale Optimization Algorithm) is used to optimize the weight parameters in the skip connections.
3. Bias loss replaces the traditional cross-entropy loss, and this loss function can optimize the errors during the feature learning process, thereby enhancing the performance of the lightweight model.
4. The proposed model is pre-trained on a plant classification task dataset, which, compared to pre-training on ImageNet, can further improve the accuracy of crop disease recognition tasks.

The rest of this paper is organized as follows: The “Related Work” section introduces and summarizes recent work related to this research; the “Materials and Methods” section describes the materials used in the experiments, relevant concepts, and the proposed method, as well as summarizes the experimental procedure; the “Experimental Results and Discussion” section includes the experimental setup and results, and evaluates and compares the experimental results with other current advanced methods; finally, the “Conclusion” section summarizes this research and proposes future research directions.

## 2 Related work

In this section, various recent works related to this study are described, and relevant methods based on machine learning and deep learning for plant disease detection are summarized. Due to the limitations of machine learning methods and the flexibility of convolutional neural networks, deep learning approaches are more common in research.

Machine learning methods have fewer parameters, shorter training cycles, and do not require a large amount of training data, making them easier to deploy in practice (Albattah et al., 2022a). However, the challenges lie in complex data preprocessing and accurate manual feature extraction processes. Sharif et al. (2018) proposed a method for automatic detection and classification of citrus diseases based on optimized weighted segmentation and feature selection. The contrast of input images is enhanced using Top-hat filters and Gaussian functions, and the weighted segmentation method using chi-square distance and threshold functions is employed to extract the enhanced lesion points. The results show that the preprocessing method further improves the accuracy of lesion segmentation. Manually extracted features still contain many noisy features, Tan Nhat et al. (2020) used Adaptive Particle-Grey Wolf metaheuristic (APGWO) to screen extracted mango leaf pathology features and combined them with artificial neural networks (ANN) to detect early mango leaf diseases. Common types of features include texture features, geometric features, statistical features, etc., and multiple features can be used in combination. Pantazi et al. (2019) used the GrabCut algorithm to segment sample images and extracted histogram features of the segmented samples using Local Binary Patterns (LBP). Li et al. (2020) employed the Gray-Level Co-occurrence Matrix (GLCM) to extract texture features from multispectral images and constructed a Binary Logistic Regression (BLR) model for cotton root rot disease classification. Experiments showed that the spectral model is suitable for more severely infected cotton fields, while the spectral-texture model is more suitable for low or moderately infected cotton fields. Different classifiers can also be combined to further improve classification accuracy, Sahu and Pandey (2023) proposed a novel hybrid Random Forest Multi-Class Support Vector Machine (HRF-MCSVM) method for plant leaf disease detection. Experimental results on PlantVillage showed that this method performs better than popular standalone classifiers.

A series of deep learning methods, represented by convolutional neural networks, have attracted widespread attention from researchers. However, their inherent dependence on high-cost computational resources limits their development space. Fortunately, in recent years, many scholars have noticed such issues and started to study the application of lightweight networks in plant disease recognition. Nandhini and Ashokkumar (2022) used the improved Henry’s Law Constant Gas Solubility Optimization algorithm to optimize the hyperparameters of the pre-trained DenseNet-121, achieving a classification accuracy of 98.7% for various plant disease classification tasks on PlantVillage. Amin et al. (2022) utilized pre-trained EfficientNetB0 and DenseNet-121 to extract deep features from corn plant images. By extracting and fusing deep features from different CNNs to generate more complex feature sets, the limitations of single lightweight CNNs in feature extraction are compensated, thereby improving classification accuracy. Zhao et al. (2022) proposed a CNN that combines Inception, residual structures, and embedded attention mechanisms, and conducted training and testing on three plant disease image datasets in PlantVillage. With a model size of 19.1 MB, they achieved a classification accuracy of 99.55%. Chen et al. (2022b) proposed an improved ResNet-18 method for disease recognition in peanut leaf datasets and PlantVillage datasets. Channel attention mechanisms were inserted into the model to enhance feature extraction capabilities, and channel pruning techniques were used to remove unimportant channels to reduce model parameters and complexity. Compared to the baseline model, the compressed model’s parameter count was reduced by 57.85%. Wang et al. (2021) formed a trilinear convolutional neural network consisting of VGG-16, InceptionV3, and ResNeXt-101 through weight sharing methods and compared the effects of no sharing, partial sharing, and complete sharing on model performance. The weight sharing mechanism can reduce the parameter count of the fused network and improve network performance. In the PlantVillage dataset test, the fully shared method based on ResNeXt-101 achieved the highest accuracy of 99.7% with 361.24M parameters. Notably, the fully shared method based on InceptionV3 had a 0.1% lower accuracy than the former but had only 91.13M parameters, seemingly having more competitive potential. Moreover, lightweight models have limited feature extraction capabilities, and in cases with fewer data samples, the network’s few-shot learning ability is more challenging. Liu and Zhang (2023) proposed an improved InceptionV3 network for few-shot learning in the plant disease diagnosis domain, achieving a prediction accuracy of 99.45% with a total of 120 training samples in four apple disease categories.

The existing research achievements of machine learning and deep learning in plant disease detection and classification are shown in **Table 1**. Although the aforementioned studies tend to favor relatively lightweight network models, it is still difficult to achieve an ideal balance between accuracy and size. These studies generally use complex networks or fused networks to achieve higher accuracy and employ network compression techniques to reduce the model’s parameter count(Wang et al., 2021; Zhao et al., 2022; Zhu et al., 2022). However, network compression is a highly challenging task, making it difficult to effectively balance accuracy and latency(Hinton et al., 2015; Garg et al., 2023).

**Table 1:** Research related to machine learning and deep learning in plant disease identification.

Reference	Method	Dataset	Accuracy
Sharif et al. (2018)	M-SVM	Citrus Diseases Image Gallery Dataset	95.8%
Tan Nhat et al. (2020)	ANN	mango leaves	85.45%
Pantazi et al. (2019)	SVM	46 plant-condition combinations	95%
Li et al. (2020)	RF、BLR	Sentinel-2 imagery	92.95%
Sahu and Pandey (2023)	HRF-MCSVM	PlantVillage	98.9%
Elaraby et al. (2022)	AlexNet	Multiple leaf	98.83%
Deng et al. (2021)	Ensemble Model	rice diseases	91%
Nandhini and Ashokkumar (2022)	DenseNet-121	PlantVillage	98.7%
Amin et al. (2022)	EfficientNetB0、DenseNet-121	corn plant leaves	98.56%
Abbas et al. (2021)	C-GAN、DensNet-121	PlantVillage	97.11%
Zhao et al. (2022)	RIC-Net	PlantVillage	99.55%

## 3 Materials and methods

### 3.1 Dataset and pre-processing

Three datasets were selected for the experiment: the PlantVillage (PV) (Hughes and Salathe, 2015), the Plant Pathology 2020 - FGVC7 (Thapa et al., 2020) apple leaf dataset, and Pl@ntNet-300K (Garcin et al., 2021). The PV dataset is popular in the plant disease classification task. It consists of healthy and diseased images of 14 crops with 38 different categories and 54,305 images. The dataset was captured in a controlled environment where the images were stripped of complex backgrounds, and only the individual leaves were retained. Therefore, the apple leaf dataset from the Plant Pathology 2020-FGVC7 Kaggle competition was used to further evaluate the model’s performance in a realistic field environment. This dataset consists of 3651 high-quality images of symptoms of multiple apple leaf diseases and contains four states of apple black star disease, cedar apple rust, multiple diseases, and healthy leaves. All images were taken in outdoor environments containing complex background conditions; each image has multiple leaves. According to the official description, this dataset has 1821 labeled images for training and testing, and the remaining unlabeled images are used to evaluate the participants’ performance. Therefore, only the 1821 annotated images from the FGVC7 Apple Leaf dataset were used as the experimental dataset in this work. The proposed model is pre-trained on Pl@ntNet-300K, a plant species dataset built from the Pl@ntNet citizen observatory database, which consists of 306,146 plant images covering 1,081 species, but excluding plant diseases.

**Figure 1:**
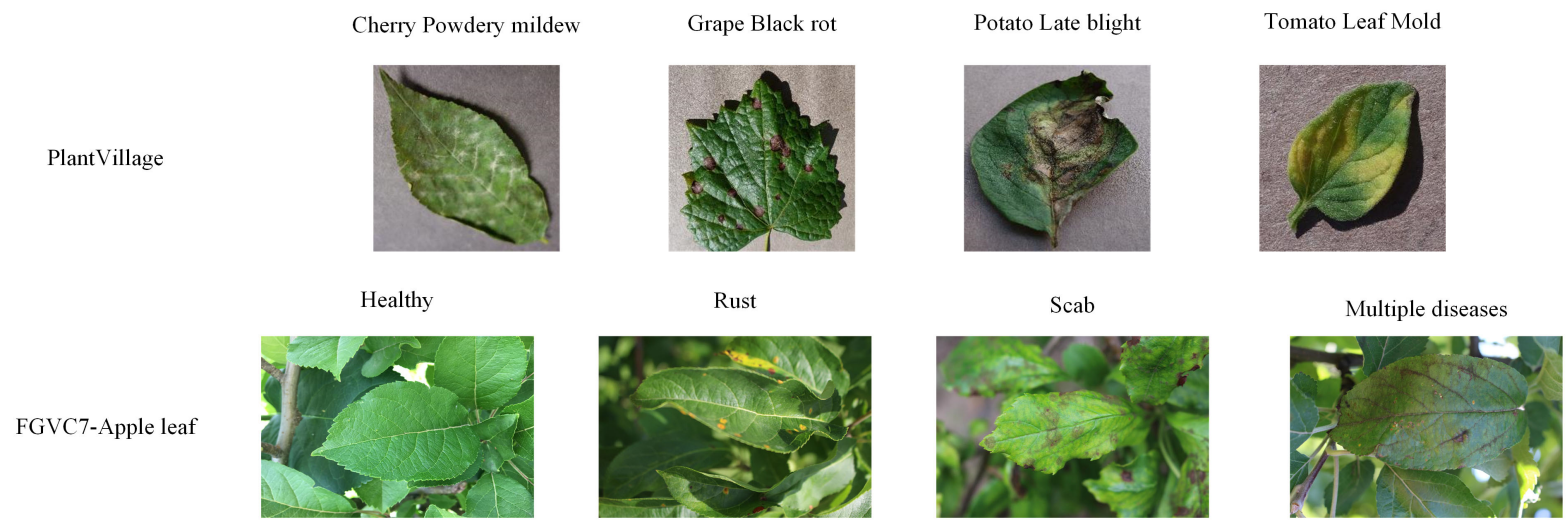
Example images of PlantVillage dataset and Plant Pathology 2020 - FGVC7 apple leaf disease dataset.

A portion of the PV dataset and the FGVC7 apple leaf dataset are shown in **Figure 1**, and the dataset was expanded using preprocessing techniques such as horizontal flipping, rotating, cropping, and resizing to prevent the model from over-fitting during training. In practical training, the image size of the apple leaf dataset is cropped to 512×512, while the PV dataset is cropped to 224×224.

### 3.2 Lightweight model

Existing lightweight models include EfficientNet, ShuffleNet, MobileNet, Xception, DenseNet, etc. On the ImageNet classification task, EfficientNet-B1 achieved 78.8% accuracy with 7.8M parameters, EfficientNet-B3 achieved 81.1% accuracy with 12M parameters (Tan and Le, 2019), MobileNet with 4.2M parameters 70.6% accuracy (Howard et al., 2017), and Xception achieved 79% accuracy with 22M number of parameters (Chollet, 2017). These deep learning models have fewer parameters and excellent performance, making them more suitable for deployment on mobile devices.

MobileNet with more balanced performance is a lightweight model designed by the Google team for mobile or embedded application scenarios, where the number of parameters and computations are reduced not only by the shallow network structure, but more importantly by using a depth-separable convolutional structure to replace the traditional standard convolutional structure.

Since some of the convolution kernels for deep convolution in MobileNetV1 may be empty after training, the Inverted Residuals structure is proposed in MobileNetV2 to solve this problem. Sandler et al., 2018 realized that when using the ReLU function, more information is lost when the dimensionality of the input features is relatively low, while more information is retained when the dimensionality of the input features is high, so the expansion layer is added to boost the input features before deep convolution. After the deep convolution is completed, a 1×1 convolution kernel is used to reduce the dimensionality of the output features. In addition, linear bottleneck have been proposed in MobileNetV2 to replace some of the ReLU with linear activation functions (Sandler et al., 2018). MobileNetV3 improves upon MobileNetV2 by renaming the basic network unit bottleneck to bneck, incorporating the squeeze-and-excite (SE) attention mechanism, utilizing Network Architecture Search (NAS) to optimize the model structure, and redesigning the time-consuming structure. In the ImageNet classification task, MobileNetV3-Large 1.0 achieved a Top-1 accuracy of 75.2% with 5.4 million parameters (Howard et al., 2019).

### 3.3 Transfer learning

**Figure 2:**
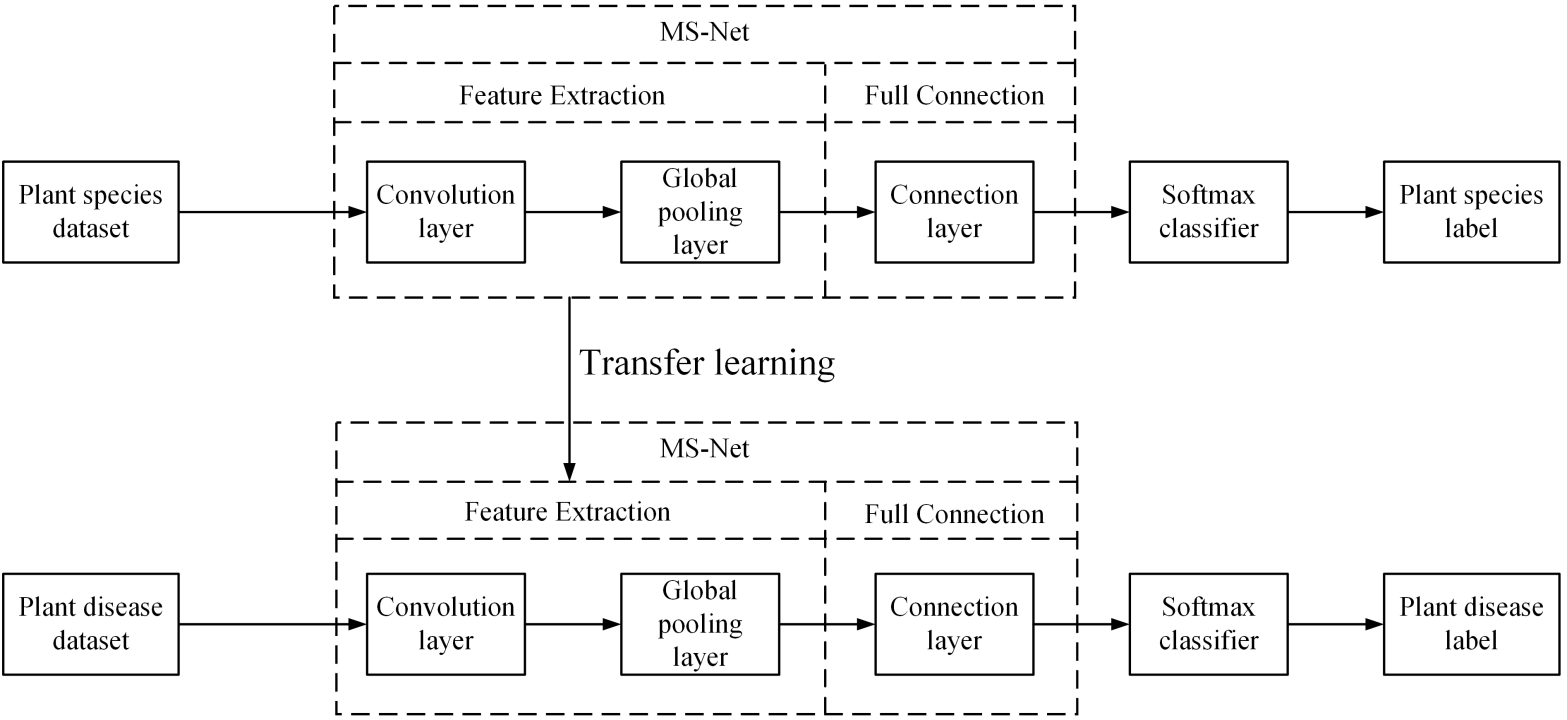
Flow chart of transfer learning of the proposed method.

Deep learning requires massive amounts of sample data to train the model, which can lead to limited model performance improvement and overfitting if the labeled dataset used to train the model is poor. However, collecting massive, labeled datasets is challenging, and manually labeling samples is time consuming and costly. Using transfer learning can solve these problems by retraining the pre-trained model from a large dataset on a small target dataset, which not only reduces the training time but also enhances the performance of the model (Chen et al., 2020; Jiang et al., 2021; Krishnamoorthy et al., 2021). In addition, Lee et al. (2020) indicated that models for plant disease classification could improve the network’s performance and reduce the effects of overfitting if they are pre-trained using plant datasets, but this approach may not apply to simpler, shallower networks. In this work, comparison experiments were conducted using pre-trained models on ImageNet and pre-trained models on Pl@ntNet-300K to verify the effectiveness of this method for the lightweight model proposed in this study. The architecture of the transfer learning workflow is shown in **Figure 2**.

### 3.4 Whale optimization algorithm

The Whale Optimization Algorithm (WOA) is a meta-heuristic optimization algorithm that finds the optimal solution by mimicking the spiral bubble net feeding of humpback whale populations in nature. The algorithm includes three types of predation behaviors of humpback whale populations: encircling prey, bubble netting to enclose prey, and randomly searching for prey. By continuously updating the position of the whale population in space through these three behaviors to achieve the search for the globally optimal solution (Mirjalili and Lewis, 2016), the algorithm has fewer parameters and is more capable of searching for the optimal global solution.

In searching for prey, the whale needs to assume the current best search agent (prey) first since the location of the prey is not known a priori, and the other search agents (whales) will update their locations to the best search agent. This behavior can be expressed as Eq. (2).


(1) $$\begin{array}{c} \vec{\text{D}}=|\text{C}.\vec{{\text{X}}^{*}}(\text{t})-\vec{\text{X}}(\text{t})| \end{array}$$

(2) $$\begin{array}{c} \vec{\text{X}}\left(\text{t}+1\right)=\vec{{\text{X}}^{*}}\left(\text{t}\right)-\vec{\text{A}}\cdot \vec{\text{D}} \end{array}$$

Where, t is the current number of iterations, $\vec{\text{A}}$ and $\vec{\text{C}}$ are the coefficient vectors, $\vec{{\text{X}}^{*}}$ is the current position of the best search agent, $\vec{\text{X}}$ is the position of the present agent, $\vec{\text{X}}\left(\text{t}+1\right)$ is the updated position, and $\vec{{\text{X}}^{*}}$ will be updated after each iteration if there is a position closer to the optimal solution. $\vec{\text{A}}$ and $\vec{\text{C}}$ are calculated using Eq. (3) and Eq. (4).


(3) $$\begin{array}{c} \vec{\text{A}}=2\vec{\text{a}}\cdot \vec{\text{r}} \end{array}$$

(4) $$\begin{array}{c} \vec{\text{C}}=2\cdot \vec{\text{r}} \end{array}$$

Where, $\vec{\text{a}}$ decreases linearly from 2 to 0 during the iteration and $\vec{\text{r}}$ is a random vector in $[0,1]$ .

In addition, whale populations also employ the bubble-net strategy to surround prey, consisting of constrictive encirclement and spiral swimming around the prey. The mathematical modeling equation for the constrictive encirclement behavior follows Eq. (2) for the prey encirclement process, but the value of $\vec{\text{A}}$ in this process is limited to $[-1,1]$ . The position search between the whale and the prey is updated using a spiral path while swimming around the prey. This can be expressed as Eq. (5).


(5) $$\begin{array}{c} \vec{\text{X}}\left(\text{t}+1\right)=\vec{{\text{D}}^{\prime }}\cdot {\text{e}}^{\text{bl}}\cdot \cos(2\pi \text{l})+\vec{{\text{X}}^{*}}\left(\text{t}\right) \end{array}$$

Where, the distance between the whale and the prey is denoted by ${\text{D}}^{\prime }=|\vec{{\text{X}}^{*}}(\text{t})-\vec{\text{X}}(\text{t})|$ , b is a constant that defines the shape of the spiral, and l is a random number in the range $\left[-1,1\right]$ . The whale has two behaviors in the process of enclosing the prey, contraction and encirclement and spiral swimming around the prey. Assuming that the probabilities of these two behaviors are equal, this process can be represented by Eq. (6).


(6) $$\begin{array}{c} \vec{\text{X}}(\text{t}+1)=\{\begin{array}{l} \vec{{\text{X}}^{*}}(\text{t})-\vec{\text{A}}\cdot \vec{\text{D}},\;\;\;\text{if p}<0.5\\ \vec{{\text{D}}^{\prime }}\cdot {\text{e}}^{\text{bl}}\cdot \cos(2\text{πl})+\vec{{\text{X}}^{*}}(\text{t}),\;\text{if p}\geq 0.5 \end{array} \end{array}$$

When the range of $\vec{\text{A}}$ does not belong to $\left[-1,1\right]$ during the contraction envelope, the humpback whale will search for its prey randomly. The current whale will choose a random whale in the whale population to approach to update its position, which will enhance the algorithm’s global search ability. The mathematical expression of this behavior can be represented by Eq. (7) and Eq. (8).


(7) $$\begin{array}{c} \vec{\text{D}}=|\vec{\text{C}}\cdot \vec{{\text{X}}_{\text{rand}}}-\vec{\text{X}}| \end{array}$$

(8) $$\begin{array}{c} \vec{\text{X}}\left(\text{t}+1\right)=\vec{{\text{X}}_{\text{rand}}}-\vec{\text{A}}\cdot \vec{\text{D}} \end{array}$$

Where, $\vec{{\text{X}}_{\text{rand}}}$ is the location of the random whale.

To further enhance the global search capability of the algorithm, the Lévy flight strategy is used to update the position of an individual whale again after it has updated its position, and the mathematical expression can be represented as Eq. (9).


(9) $$\begin{array}{c} \vec{\text{X}}\left(\text{t}+1\right)=\vec{\text{X}}\left(\text{t}\right)+0.01\cdot \frac{\vec{\text{u}}}{{\left|\vec{\text{v}}\right|}^{\frac{1}{\text{β}}}}\cdot \vec{\text{X}}\left(\text{t}\right) \end{array}$$

Where, β takes values in the range $\left(0,2\right)$ , β=1.5 in this work, each component of $\vec{\text{u}}$ and $\vec{\text{v}}$ follows the normal distribution as described in Eq. (10) and Eq. (11).


(10) $$\begin{array}{c} {\text{u}}_{\text{d}}∼\text{N}\left(0,\sigma_{\text{u}}^{2}\right),\;{\text{v}}_{\text{d}}∼\text{N}\left(0,\sigma_{\text{v}}^{2}\right) \end{array}$$

(11) $$\begin{array}{c} {\text{σ}}_{\text{u}}={\left\{\frac{\text{Γ}(1+\text{β})\times \sin(\frac{\text{πβ}}{2})}{\text{β}\times \text{Γ}(\frac{1+\text{β}}{2})\times 2^{\frac{\text{β}-1}{2}}}\right\}}^{\frac{1}{\text{β}}},\;{\text{σ}}_{\text{v}}=1 \end{array}$$

**Figure 3:**
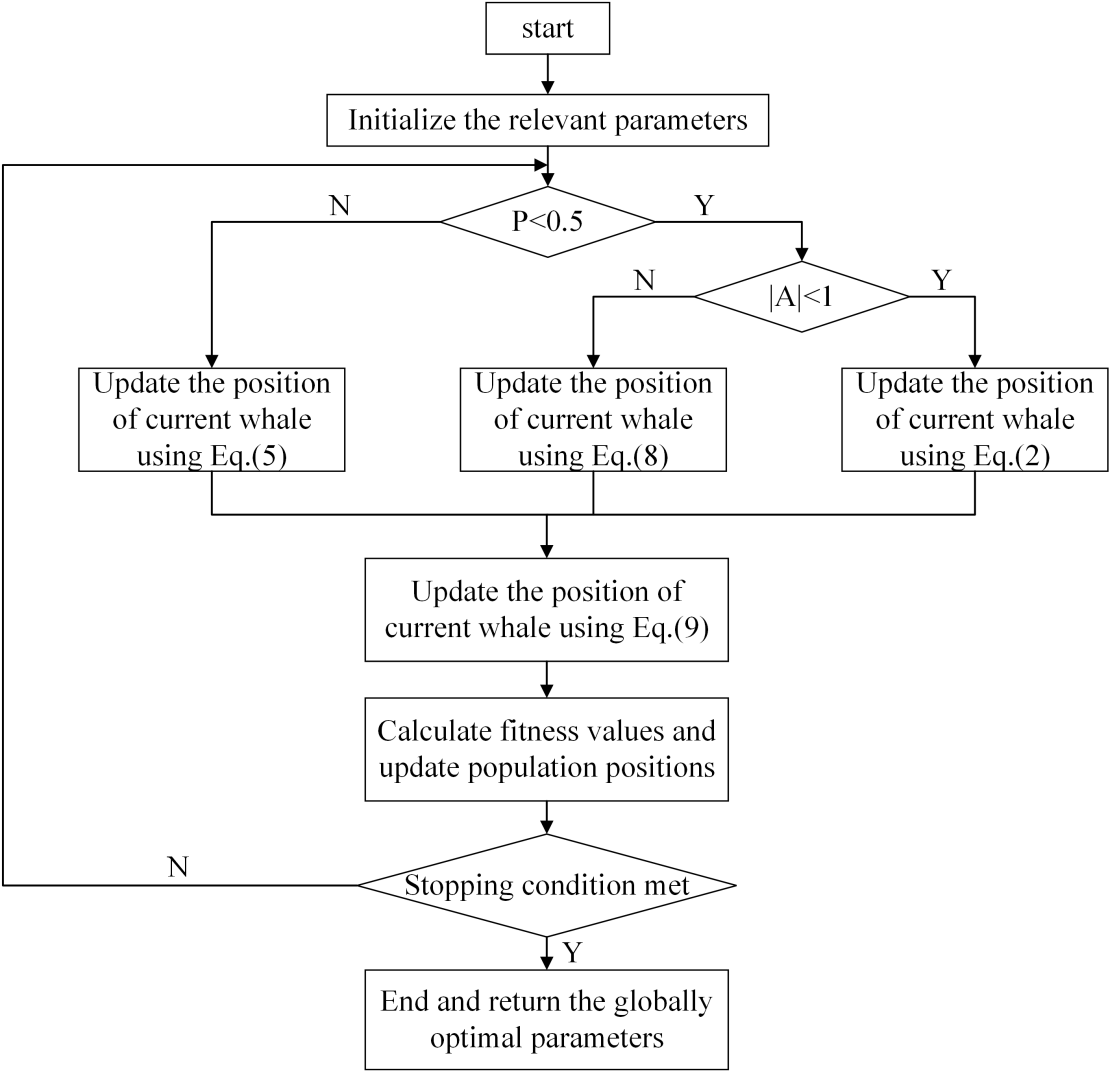
Flowchart of the Whale Optimization Algorithm.

Where, ${\text{u}}_{\text{d}}$ denotes the component of $\vec{\text{u}}$ and ${\text{v}}_{\text{d}}$ denotes the component of $\vec{\text{v}}$ . The process of the whale optimization algorithm is demonstrated in **Figure 3**.

**Figure 4:**
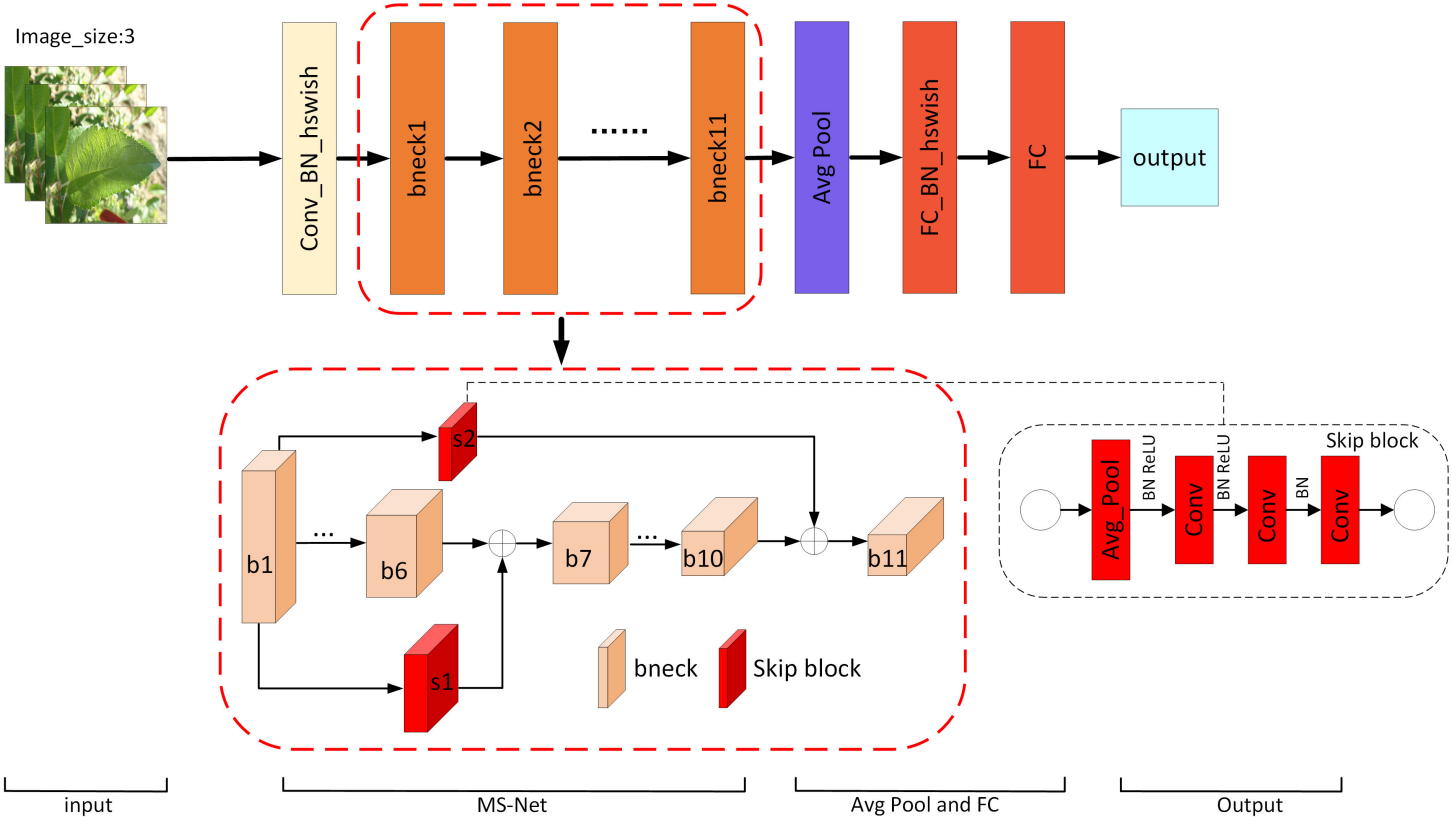
The proposed MS-Net architecture.

Two skip blocks, s1 and s2, are embedded in the proposed network architecture as shown in **Figure 4**. To achieve better fusion, different weights are assigned to the input feature maps of each jump connection and the weight parameters are automatically adjusted using the whale optimization algorithm.

The mathematical expressions for the input features of the bneck7 and bneck11 network base units are as follows.


(12) $$\begin{array}{c} {\text{F}}_{\text{b}7}^{\text{in}}={\text{w}}_{1}\cdot {\text{F}}_{\text{s}1}^{\text{out}}+{\text{w}}_{2}\cdot {\text{F}}_{\text{b}6}^{\text{out}} \end{array}$$

(13) $$\begin{array}{c} {\text{F}}_{\text{b}11}^{\text{in}}={\text{w}}_{3}\cdot {\text{F}}_{\text{s}2}^{\text{out}}+{\text{w}}_{4}\cdot {\text{F}}_{\text{b}10}^{\text{out}} \end{array}$$

Where, ${\text{F}}^{\text{in}}$ denotes the input features, ${\text{F}}^{\text{out}}$ denotes the output features, ${\text{w}}_{\text{i}}$ denotes the weight parameters of different feature maps and satisfies ${\text{w}}_{1}+{\text{w}}_{2}=1$ , ${\text{w}}_{3}+{\text{w}}_{4}=1$ .

### 3.5 Proposed approach

Considering the superior performance of MobileNetV3 with the inclusion of the SE attention mechanism and optimized with NAS, MobileNetV3-Small is used as the feature extraction network in this work. The small version of the feature extraction network consists of 11 bnecks, which has fewer parameters compared to the large version, but the performance is also degraded. In this paper, the classical MobileNetV3-Small is modified by adding two skip blocks of different sizes after the first bneck to pass the extracted low-level features to the 7th and 11th bneck, enriching their input feature information and thus improving the classification performance of the model. Abrahamyan et al. (2021) proposed skip block to enhance the performance of compact CNNs. To achieve skip connectivity in the network, adaptive average pooling operation and convolution operation are used in the skip block to reduce the spatial size of feature information and retain key features (Ahmed et al., 2022). In addition, to achieve better feature fusion, different weights are assigned to the input feature maps of the skip connected parts, and the whale optimization algorithm is used to search for globally optimal parameters. The newly generated network model is called MS-Net, and the network structure is shown in **Figure 4**.

Abrahamyan et al. (2021) note that in compact CNNs, the limited number of parameters always makes it unlikely for the model to obtain rich features, and some irrelevant and redundant data may negatively affect the optimization process of the model and affect the final performance. There is no way to avoid this effect in the standard cross-entropy loss function, which gives equal weight to all data, and the standard cross-entropy loss is mathematically defined by Eq. (14).


(14) $$\begin{array}{c} {\text{L}}_{\text{ce}}=-\frac{1}{\text{N}}\sum_{\text{i}=1}^{\text{N}}\sum_{\text{j}=1}^{\text{k}}{\text{y}}_{\text{ij}}\log\left({\text{p}}_{\text{ij}}\right) \end{array}$$

Where, N represents the number of samples, and k represents the number of categories. ${\text{p}}_{\text{ij}}$ represents the probability that sample i belongs to category j . ${\text{y}}_{\text{ij}}$ is a one-hot encoding; if sample i belongs to category j , then the value of ${\text{y}}_{\text{ij}}$ is 1, otherwise, it is 0.

Abrahamyan et al. (2021) proposed bias loss to mitigate this negative consequence. The variance is applied to measure the feature diversity contained in the sample data and to weight each data point to prevent samples with poor feature diversity from influencing the optimization process. The mathematical representation of bias loss is given by Eq. (15)-Eq. (18).


(15) $$\begin{array}{c} {\text{L}}_{\text{bias}}=-\frac{1}{\text{N}}\sum_{\text{i}=1}^{\text{N}}\sum_{\text{j}=1}^{\text{k}}\text{z}\left({\text{v}}_{\text{i}}\right){\text{y}}_{\text{ij}}\log\left({\text{p}}_{\text{ij}}\right) \end{array}$$

(16) $$\begin{array}{c} \text{z}\left({\text{v}}_{\text{i}}\right)=\exp\left({\text{v}}_{\text{i}}*\text{α}\right)-\text{β} \end{array}$$

(17) $$\begin{array}{c} {\text{v}}_{\text{i}}=\frac{\sum_{\text{j}=1}^{\text{n}}{\left({\text{t}}_{\text{j}}-\text{μ}\right)}^{2}}{\text{n}-1} \end{array}$$

(18) $$\begin{array}{c} \text{μ}=\frac{\sum_{\text{j}=1}^{\text{n}}{\text{t}}_{\text{j}}}{\text{n}} \end{array}$$

Where, α and β are adjustable contribution parameters, which can generally be set to α=0.3 and β=0.3 . The variable ${\text{v}}_{\text{i}}$ represents the proportional variance of the output characteristics of the ith data point in the batch. The $\text{t}\in {\text{R}}^{\text{b}\times \text{n}}$ denotes the output of the convolutional layer, while b stands for the batch size. Additionally, n=c×h×w , where c corresponds to the number of input channels, and h and w represent the tensor width and height, respectively.

In this paper, bias loss is used to replace the conventional cross-entropy loss to minimize the impact of redundant data in the samples on MS-Net performance. Based on the transfer learning approach, the proposed network model was first pre-trained on the plant species dataset Pl@ntNet-300K, and then the completed pre-trained model was fine-tuned on the PlantVillage dataset and the FGVC7 apple leaf dataset.

## 4 Results and discussion

### 4.1 Experimental setup

To fully evaluate the model’s performance, experiments were conducted on the PlantVillage and the FGVC7 Apple leaf datasets, and the following quality metrics: Accuracy, Precision, Recall, F1-score (F1), and confusion matrix were used. Where Accuracy is the percentage of correctly predicted samples out of the total samples, Precision is the probability of being genuinely positive out of all samples predicted to be positive, and Recall is the probability of being predicted to be positive out of the genuinely positive samples, and F1-score is a combined measure of Accuracy and Recall. The mathematical definitions of these metrics as Eq. (19)-Eq. (22).


(19) $$\begin{array}{c} \text{Accuracy}=\frac{\text{TN}+\text{TP}}{\text{TN}+\text{TP}+\text{FN}+\text{FP}} \end{array}$$

(20) $$\begin{array}{c} \text{Precision}=\frac{\text{TP}}{\text{TP}+\text{FP}} \end{array}$$

(21) $$\begin{array}{c} \text{Recall}=\frac{\text{TP}}{\text{FN}+\text{TP}} \end{array}$$

(22) $$\begin{array}{c} F1-\text{score}=\frac{2\text{TP}}{2\text{TP}+\text{FN}+\text{FP}} \end{array}$$

Where TP, TN, FP, and FN represent true positive, true negative, false positive, and false negative, respectively.

The experimental platform used in this research: the hardware environment was Intel(R) Xeon(R) Silver 4314 CPU 2.40GH, 64G RAM, NVIDIA GeForce RTX 3090 GPU; the software environment was Ubuntu 20.04 system, Python3.9, and PyTorch1.11.0.

### 4.2 Experiments on the FGVC7

**Figure 5:**
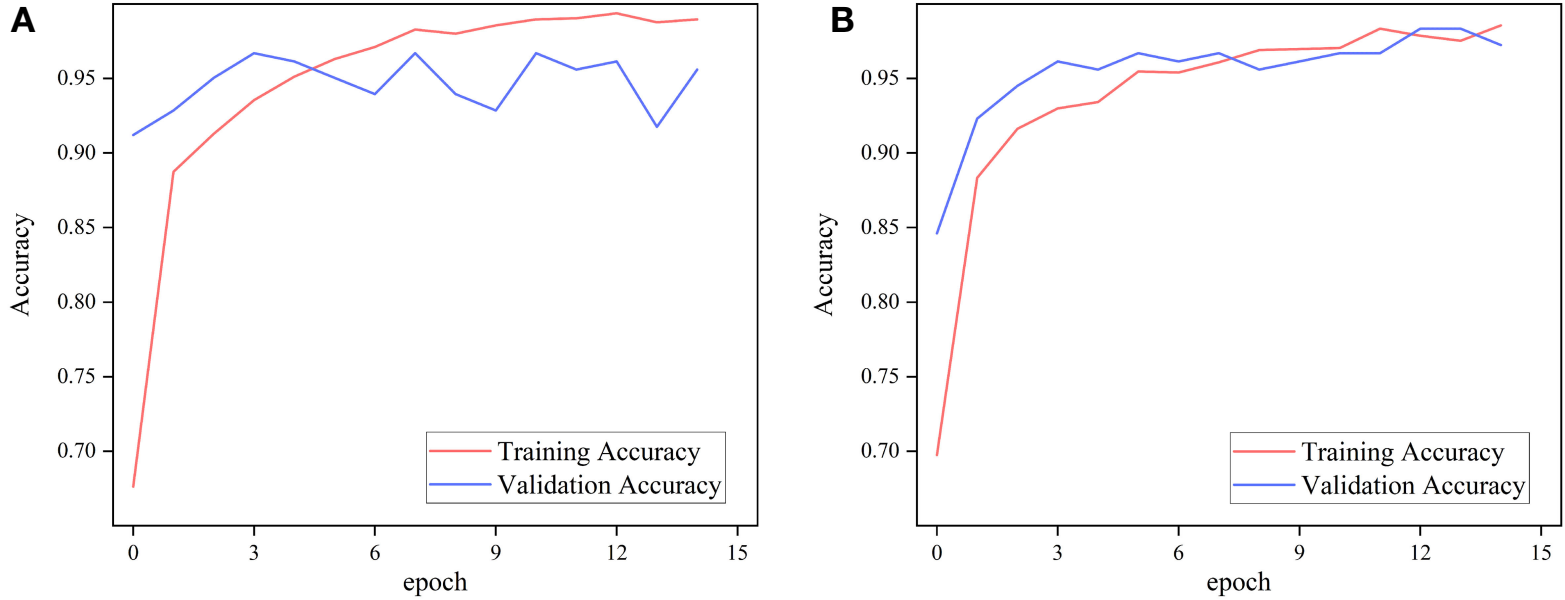
Performance of MobileNetv3 on FGVC7 apple leaf disease dataset using different pre-training methods. **(A)** ImageNet and **(B)** Pl@ntNet-300K.

To evaluate the performance of the ImageNet pre-trained model and the Pl@ntNet-300K pre-trained model on the FGVC7 apple leaf dataset with a realistic field background, two pre-training schemes of MobileNetV3 Small were used for ablation experiments. To approximate the 1,000 classes found in the ImageNet dataset, a random selection of classes was excluded from the Pl@ntNet-300K dataset, resulting in a total of 966 classes. These classes were then divided into training and validation sets in a 4:1 ratio. The pre-trained model was run for 15 epochs on the FGVC7 apple leaf dataset, **Figure 5** depicts the training performance of MobileNetV3 utilizing the two pre-training methods on the apple leaf dataset with a realistic field background, and **Table 2** summarizes the performance of the models with different pre-training approaches on the test set.

**Table 2:** Recognition results under two pre-training methods.

Pre-train dataset	Class	Precision	Recall	F1-score	Support
ImageNet	healthy	0.909	0.980	0.943	52
	multiple	1.000	0.333	0.500	9
	rust	0.968	0.984	0.976	62
	scab	0.950	0.966	0.958	59
	weighted avg	0.947	0.945	0.937	182
	accuracy	0.945			182
Pl@ntNet-300K	healthy	0.962	0.980	0.971	52
	multiple	0.800	0.444	0.571	9
	rust	0.984	0.984	0.984	62
	scab	0.952	1.000	0.975	59
	weighted avg	0.958	0.961	0.957	182
	accuracy	0.961			182

**Figure 6:**
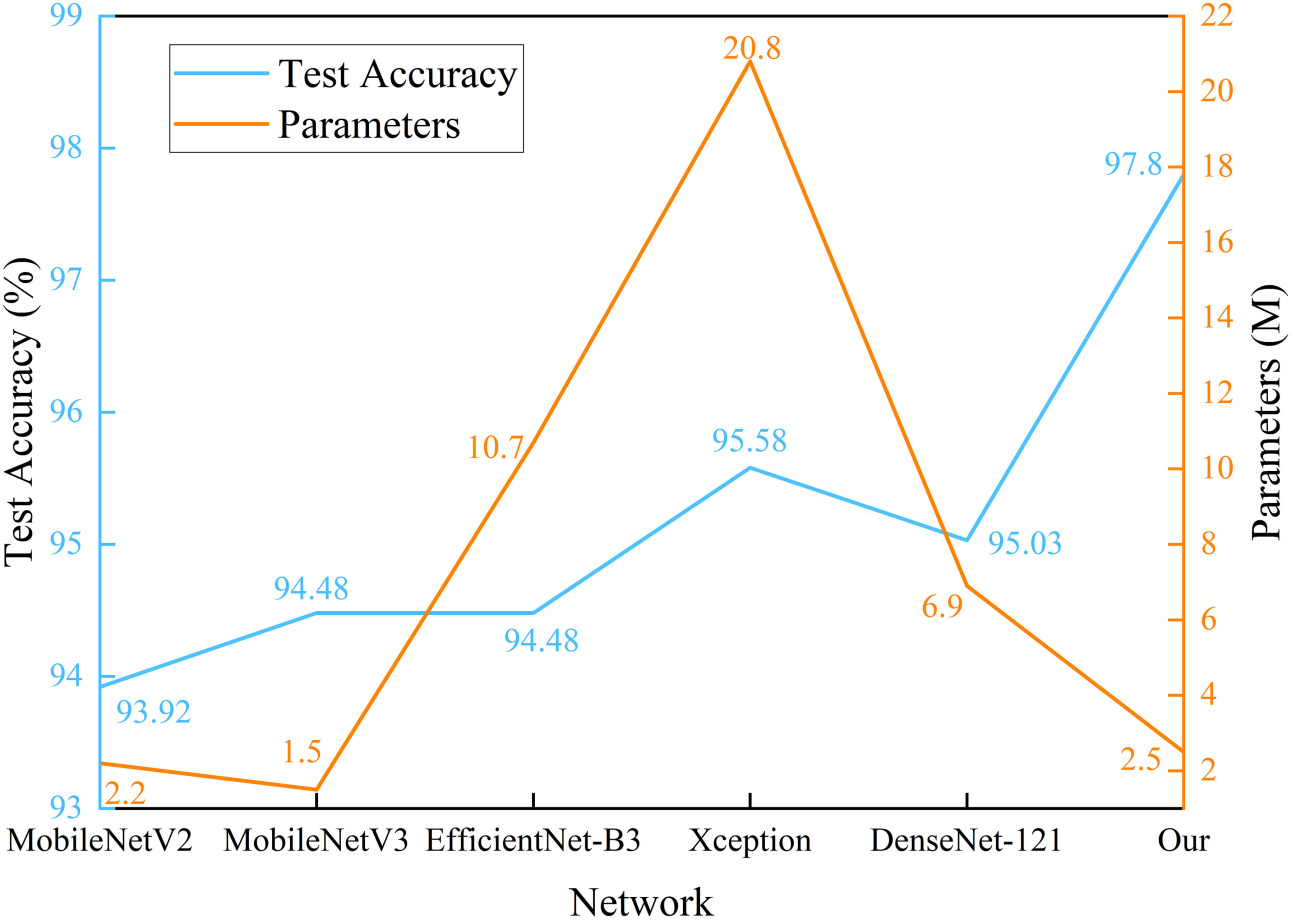
Parameters and test accuracy of identification models for apple disease identification.

**Figure 7:**
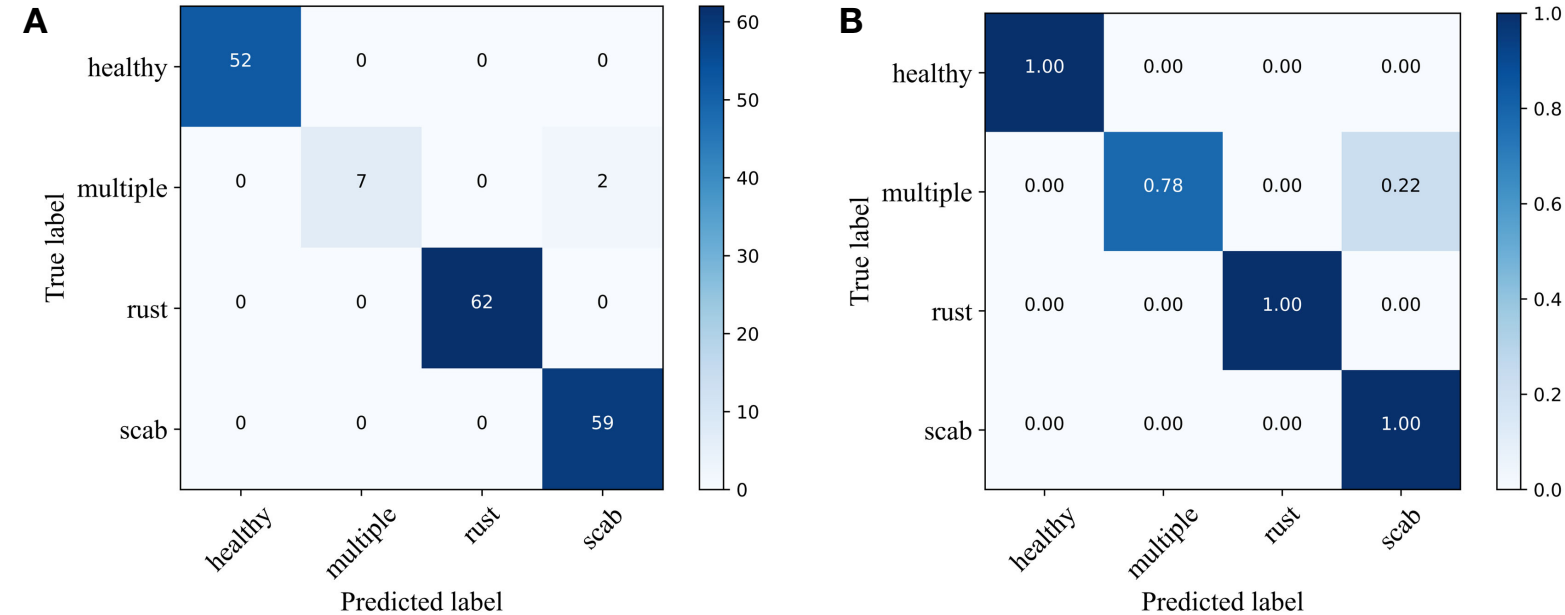
Confusion matrix of different apple diseases. **(A)** number of classes and **(B)** probabilities of classes.

The experimental results show that for classifying multiple apple leaf diseases with a realistic background in the field, the accuracy of the model pre-trained using ImageNet is 94.47% on the test set, while the pre-trained model on Pl@ntNet-300K achieves an accuracy of 96.13%. The model pre-trained with Pl@ntNet-300K outperforms the model pre-trained with ImageNet, improving the accuracy by 1.66%. **Figure 5** also illustrates that the model pre-trained with Pl@ntNet-300K has better data convergence and fit during the training process compared to the model pre-trained with ImageNet. The reason for the better pre-training results on the plant classification task dataset may be that utilizing datasets within similar domains can provide richer feature information for the compact CNN during pre-training compared to the more broadly generalized ImageNet dataset, allowing the model to learn more features about similar target tasks. Consequently, the proposed MS-Net will be pre-trained on Pl@ntNet-300K and then fine-tuned on the FGVC7 apple leaf dataset. To further evaluate the performance of the proposed method on the FGVC7 apple leaf dataset, MobileNetV2, MobileNetV3, EfficientNet-B3, Xception, and DenseNet-121 are used to perform comparative experiments on the FGVC7 apple leaf dataset. All networks were obtained pre-trained weights from ImageNet and trained for 15 epochs. **Figure 6** depicts the performance of the proposed method compared to other lightweight models. **Table 3** summarizes the test accuracy, F1 score, parameter count, FLOPs, memory size, and training time for all models. **Table 4** s shows the prediction results of the models on the test set, and **Figure 7** presents the confusion matrix of the models on the test set.

**Table 3:** Experimental results of the proposed method and existing models on the Apple dataset.

Method	Test Accuracy (%)	F1-score (%)	Parameters (M)	GFLOPs	Size(MB)	Time (h)
MobileNetV2	93.92	92.77	2.2	25.0	8.74	00:07:45
MobileNetV3	94.48	93.73	1.5	5.04	5.93	00:07:44
EfficientNet-B3	94.48	93.78	10.7	80.2	41.3	00:07:51
Xception	95.58	94.40	20.8	386.3	79.6	00:07:53
DenseNet-121	95.03	94.10	6.9	236.8	27.1	00:08:00
Proposed method	97.80	97.65	2.5	12.8	9.80	00:07:48

**Table 4:** The recognition results of different apple diseases.

Class	Precision	Recall	F1-score	Support
healthy	0.963	1.000	0.981	52
multiple	1.000	0.667	0.800	9
rust	1.000	1.000	1.000	62
scab	0.967	0.983	0.975	59
weighted avg	0.979	0.978	0.977	182
accuracy	0.978			182

From **Figure 6**, it can be observed that the proposed method has superior performance. Meanwhile, as shown in **Table 3**, after 15 epochs, the method proposed in this paper achieves the best accuracy with fewer parameters, FLOPs, and memory size. Compared to the unimproved original MobileNetV3 network, the method proposed in this paper slightly increases network complexity but achieves a significant improvement in accuracy, with almost the same training time and no significant increase in memory size. In addition, most baseline models have relatively large FLOPs, and the baseline models chosen in this study are popular lightweight networks. The reason for this phenomenon is that the dataset size is relatively large. The original pixel size of the apple leaf dataset is 2048×1365. To preserve image features as much as possible, we resize it to 512×512, but this still brings a considerable amount of computational overhead. It is worth noting that even though all models have significant complexity differences, there is no noticeable difference in the time consumed by all models when training for only 15 epochs. If the training cycles are increased, the differences in the time consumed by the models will be further magnified. As can be seen from **Table 4** and **Figure 7**, the classification results of the “multiple” class have a significant impact on the quality indicators of the model. Analysing the dataset reveals that this phenomenon is caused by the uneven distribution of categories in the FGVC7 apple leaf dataset. Among the 1821 images, there are only 91 in the “multiple” class. The limited number of training samples and the presence of multiple disease features always constrain the performance improvement of the model. If the amount of data for this class is increased or some advanced data augmentation methods (such as Generative Adversarial Networks) are used to expand the multi-disease category dataset, the overall performance of the model can be further improved.

### 4.3 Experiments on the PlantVillage

**Figure 8:**
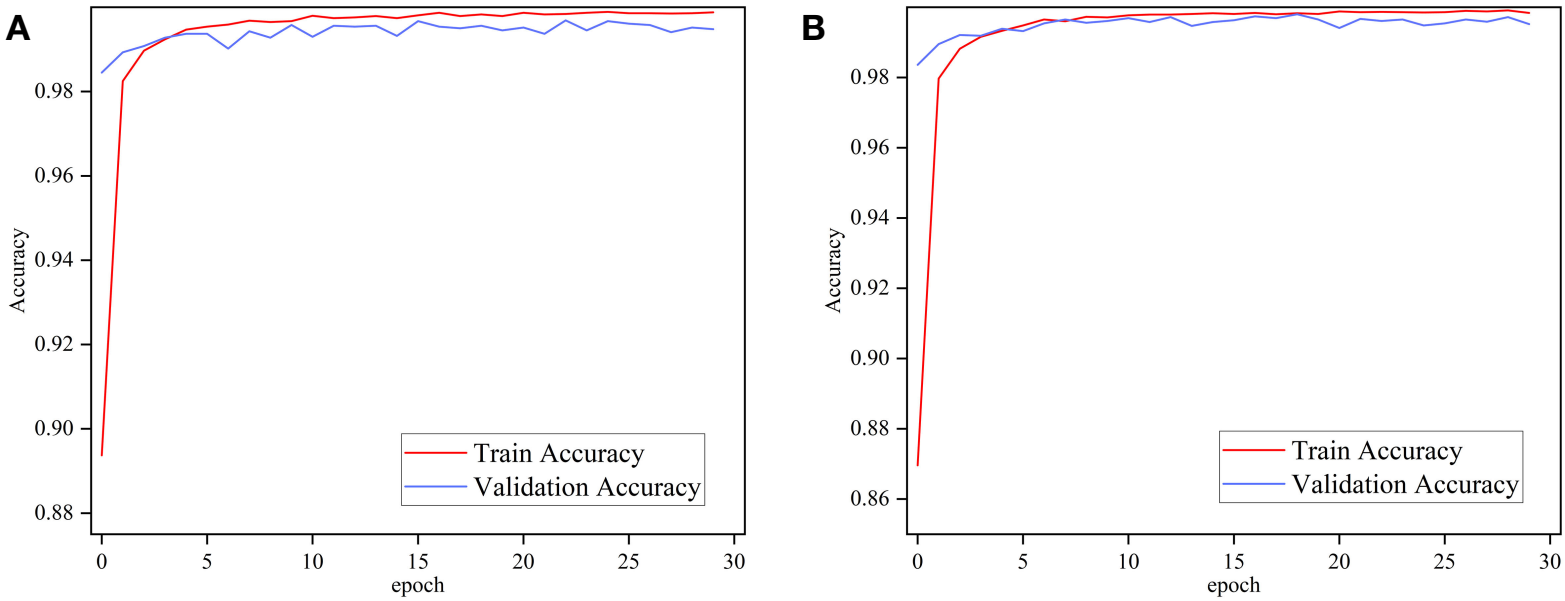
Performance of MobileNetv3 on PlantVillage dataset using different pre-training methods. **(A)** ImageNet and **(B)** Pl@ntNet-300K.

To test the performance of the proposed method under different disease conditions in different crops and to compare it with other state-of-the-art methods, the proposed method is verified in this work on the publicly available PlantVillage dataset. Ablation experiments were performed using ImageNet and Pl@ntNet-300K pre-trained MobileNetV3 Small to verify whether transfer learning in similar domains is effective on the PlantVillage dataset. The two pre-trained models were trained for 30 epochs each, and **Figure 8** depicts the performance of the two models on the PlantVillage dataset.

**Figure 8** demonstrates that the performance difference between MobileNetV3 pre-trained with ImageNet and Pl@ntNet-300K is minimal. The pre-trained networks using these two methods achieve 99.65% and 99.76% classification accuracy on the test set, respectively. The transfer learning method using similar domains on the FGVC7 Apple Leaf dataset exhibited significant performance gains, the reason for which is attributed to the fact that the two datasets are too different. As shown in **Figure 1**, the PlantVillage dataset was captured under controlled conditions without complex backgrounds and multiple leaves, and the images contained only individual plant leaves, so the models achieved similar performance in both pre-training conditions. Comparison of the aforementioned work leads to the conclusion that transfer learning on similar domain datasets enhances the robustness of the model and can further improve the performance of plant disease diagnostic models.

**Figure 9:**
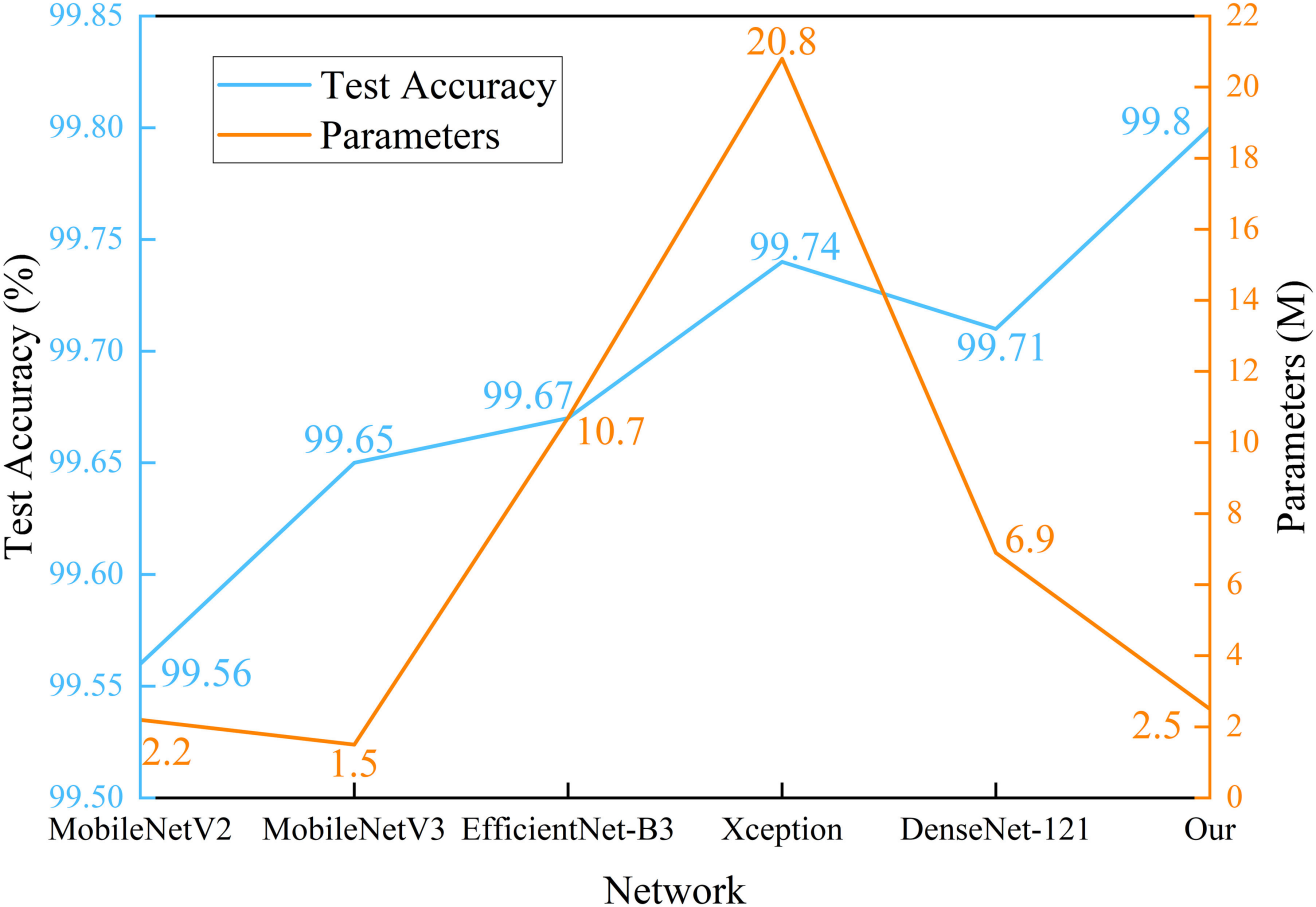
Parameters and test accuracy of multiple disease identification models.

To evaluate the performance of the method proposed in this paper on PlantVillage, we also conducted comparative experiments with the other five lightweight models mentioned earlier, which obtained pre-trained weights from ImageNet. All networks were trained for 30 epochs, and the performance of each network is shown in **Figure 9**. **Table 5** summarizes the accuracy, parameter count, F1 score, FLOPs, memory size, and training time of all models on the PlantVillage dataset.

**Table 5:** Experimental results of the proposed method and existing models on the PlantVillage dataset.

Method	Test Accuracy (%)	F1-score (%)	Parameters (M)	GFLOPs	Size(MB)	Time (h)
MobileNetV2	99.56	99.56	2.2	9.58	8.90	00:57:25
MobileNetV3	99.65	99.65	1.5	1.95	6.06	00:57:00
EfficientNet-B3	99.67	99.67	10.7	30.76	41.5	01:39:19
Xception	99.74	99.74	20.8	147.1	79.9	01:13:47
DenseNet-121	99.71	99.69	6.9	90.6	27.4	01:57:25
Proposed method	99.80	99.80	2.5	2.47	9.93	01:00:57

From **Figure 9** and **Table 5**, the method proposed in this paper performs better when considering both performance and parameter count. After 30 epochs of training, the proposed method achieves the best accuracy of 99.80%. It can be observed that MobileNetV3, with the smallest parameter count, has the shortest training time. Comparing MobileNetV2 and the proposed method, it can be concluded that the impact of small changes in parameter count on training time is almost negligible. However, DenseNet-121, which also has a relatively low parameter count, takes the longest training time. The reason is that this network has a larger number of FLOPs, resulting in a high computational load. In the design of compact CNNs, not only the parameter count of the network should be considered, but also the computational complexity of the network should be given attention. Interestingly, Xception, which has more parameters and FLOPs, has a shorter training time than DenseNet-121. The reason is that DenseNet-121 uses standard convolution, while Xception uses depthwise separable convolution, which reduces the number of multiplications and additions required, thus shortening the training time. Furthermore, it can be seen that the difference in test accuracy between the method proposed in this paper and other lightweight baseline models is not significant, and almost all models achieve excellent test accuracy on the PV dataset. As we mentioned earlier, the PV dataset was created in a laboratory environment, with each sample image having a complex background removed and centered in the frame, which also results in a high similarity in the distribution of samples within the same class in the dataset. This is precisely why we want to test our method on the apple dataset, which has a more complex outdoor background and stronger random distribution. Combined with **Table 3**, our method has stronger robustness and achieves the best prediction accuracy in field tests. In the proposed method, due to the addition of skip connections, the higher layers of the network obtain richer features with a smaller increase in network parameters. Using the bias function instead of the traditional cross-entropy loss function further reduces the impact of redundant features on compact networks during the learning process.

**Table 6** summarizes the existing research results on the PlantVillage dataset. Compared to other current advanced methods, the proposed method achieves the highest accuracy of 0.998 and performs equally well in other evaluation metrics characterizing lightweight models. Among them, the performance of CACPNET is closest to our method. CACPNET further reduces the model’s complexity and memory size based on channel pruning (Chen et al., 2022b). Channel pruning is a highly challenging task that requires calculating the weights of each channel and sorting them, as well as a certain degree of manual adjustment to achieve the desired performance. Moreover, CACPNET has the longest training cycles among all methods. Other methods are trained for about 30 epochs, while CACPNET requires 200 epochs of training. Our method can be simply understood as expanding based on a small model, with easy operations and the ability to easily generalize to other smaller lightweight models. In addition, the T-CNN model achieves similar classification accuracy with a much larger parameter count. The reason is that integrating multiple models can indeed easily improve classification accuracy, but at the same time, it also increases the overall parameter count of the model (Wang et al., 2021). It is worth noting that although the accuracy of the L-CSMS model is not as high as other methods, the resource consumption of this network is extremely low, making it a more viable option in extreme situations (Xiang et al., 2021).

**Table 6:** Comparison with other current state-of-the-art methods in the literature.

literature	Model	Parameters (M)	GFLOPs	Size(MB)	Accuracy (%)
Mohanty et al. (2016)	GoogleNet	5.0	–	–	99.35
Wang et al. (2021)	T-CNN	91.1	–	–	99.60
Xiang et al. (2021)	L-CSMS	**0.79**	0.12	–	97.90
Thakur et al. (2022)	VGG-ICNN	6	45.7	23.2	99.16
Chen et al. (2022b)	CACPNET	4.7	1.267	18.0	99.70
This study	MS-Net	2.5	2.47	**9.93**	**99.80**

The bold values means that they have achieved the best performance metric results.

## 5 Conclusion

The research on lightweight models with fewer parameters, lower complexity, and higher accuracy can further promote the popularization of automatic crop disease diagnosis methods. This study proposes a novel lightweight convolutional neural network for plant disease recognition, which has low parameter count and high accuracy. This is achieved by embedding skip blocks in the front end of the feature extraction network and optimizing the weight parameters in the skip connections using the improved whale algorithm. Bias loss replaces the traditional cross-entropy loss, reducing the negative impact of redundant data in limited features on the model learning process. At the same time, pre-training the proposed model on a plant species dataset further enhances the model’s performance and robustness. Experimental results show that, compared to the traditional transfer learning method, the proposed pre-training method improves the prediction accuracy on the apple leaf dataset by 1.6%. Compared to the original model, the prediction accuracy of the proposed method is increased by only 3.32% and 0.15% on the FGVC7 apple leaf dataset and PlantVillage dataset, respectively. The method proposed in this paper has strong robustness and achieves better performance on the apple leaf dataset with complex outdoor backgrounds, reaching the highest test accuracy with lower resource requirements. Compared to recent advanced techniques, the method proposed in this paper has lower parameter count, FLOPs, memory size, and higher recognition accuracy. Our research is beneficial for plant disease diagnosis in resource-constrained scenarios, low-resource, high-accuracy models can reduce the cost of hardware equipment and promote the development of automatic crop disease diagnosis solutions in the agricultural field. It should be noted that our method still has some shortcomings. Compared to other advanced methods, the FLOPs performance of the model is not outstanding. For the future research, we plan to analyse the training efficiency of the model, reduce the computational resources of the network, and develop a portable handheld device for plant disease diagnosis, deploying the proposed model on the device for practical applications in automatic plant disease diagnosis scenarios.

## Data availability statement

Publicly available datasets were analyzed in this study. This data can be found here: https://zenodo.org/record/5645731#.YeGDOdvjKWh (Pl@ntNet-300K), https://github.com/spMohanty/PlantVillage-Dataset/tree/master/raw/color (PlantVillage), https://www.kaggle.com/competitions/plant-pathology-2020-fgvc7/data (FGVC7 Apple Leaf).

## Author contributions

JW: Conceptualization, Funding acquisition, Writing – review & editing. SQ: Conceptualization, Methodology, Supervision, Visualization, Writing – original draft. ZJ: Methodology, Supervision, Writing – review & editing. MY: Investigation, Methodology, Software, Writing – review & editing. QX: Data curation, Investigation, Methodology, Writing – review & editing.
